# Supplementary material for: The Effect of α-Fe2O3(0001) Surface Containing Hydroxyl Radicals and Ozone on the Formation Mechanism of Environmentally Persistent Free Radicals
Source: Toxics. 2024 Aug 10;12(8):582. doi: 10.3390/toxics12080582 (PMC11359140; doi:10.3390/toxics12080582)
Supplement: Supplementary file 1 [file toxics-12-00582-s001.zip › toxics-3114689-supplementary.pdf]

Supporting Information to

The Effect of α-Fe_2O_3(0001) Surface Containing Hydroxyl Radicals and Ozone on the Formation Mechanism of Environmentally Persistent Free Radicals

Danli Liang[1], Jiarong Liu[1,2*], Chunlin Wang,[2] Kaipeng Tu[1,3], Li Wang[1,4], Lili Qiu[1], Xiuhui Zhang[1], Ling Liu[1*]

[1]Key Laboratory of Cluster Science, Ministry of Education of China, School of Chemistry and Chemical Engineering, Beijing Institute of Technology, Beijing, 100081, China

[2]Key Laboratory of National Land Space Planning and Disaster Emergency Management of Inner Mongolia, School of Resources, Environment and Architectural Engineering, Chifeng University, Chifeng, 024000, China

[3]State Key Laboratory for Structural Chemistry of Unstable and Stable Species, CAS Research/Education Center for Excellence in Molecular Sciences, Institute of Chemistry, Chinese Academy of Sciences, Beijing 100190, China.

[4]Norinco Group Shanxi North Xingan Chemical Industry Company Limited, Taiyuan 030008, China

*E-mail: lingliu@bit.edu.cn, Jiarongliu2017@163.com

Contents

Section 1. Figures..S2

Section 1. Figures

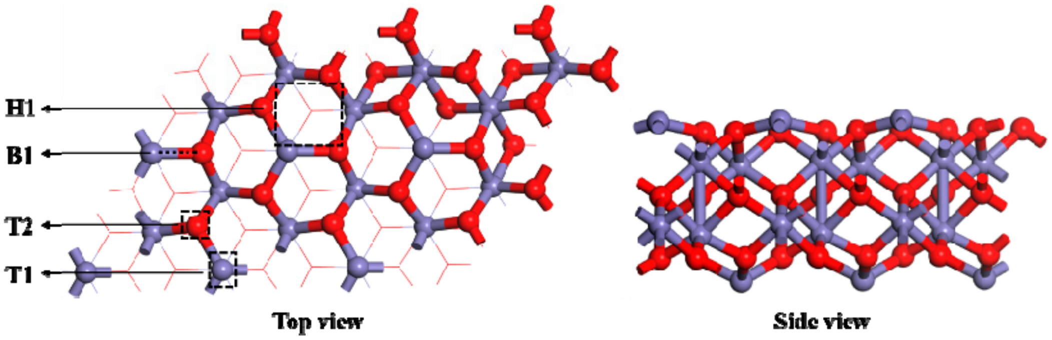

Figure S1 The top and side views of surface morphology and the corresponding adsorption sites on the α-Fe_2O_3(0001) surface. Color code: Fe (blue), O (red).

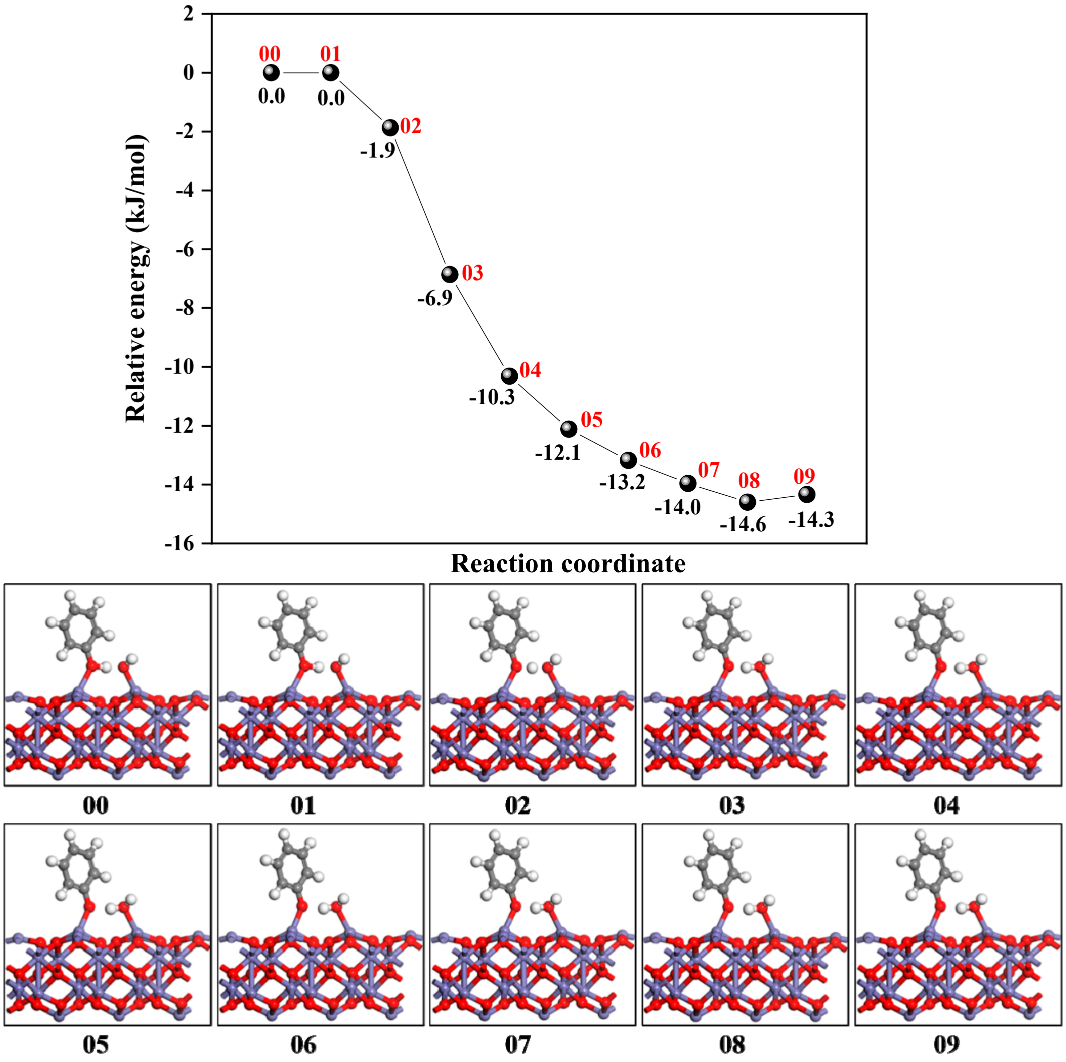

Figure S2 The energy profile for the reaction of C_6H_5OH on the α-Fe_2O_3(0001) surface containing ·OH together with the corresponding structures in one-to-one correspondence with the black points in Figure 1. Energy is in kJ/mol. Color code: Fe (blue), C (gray), O (red), and H (white).

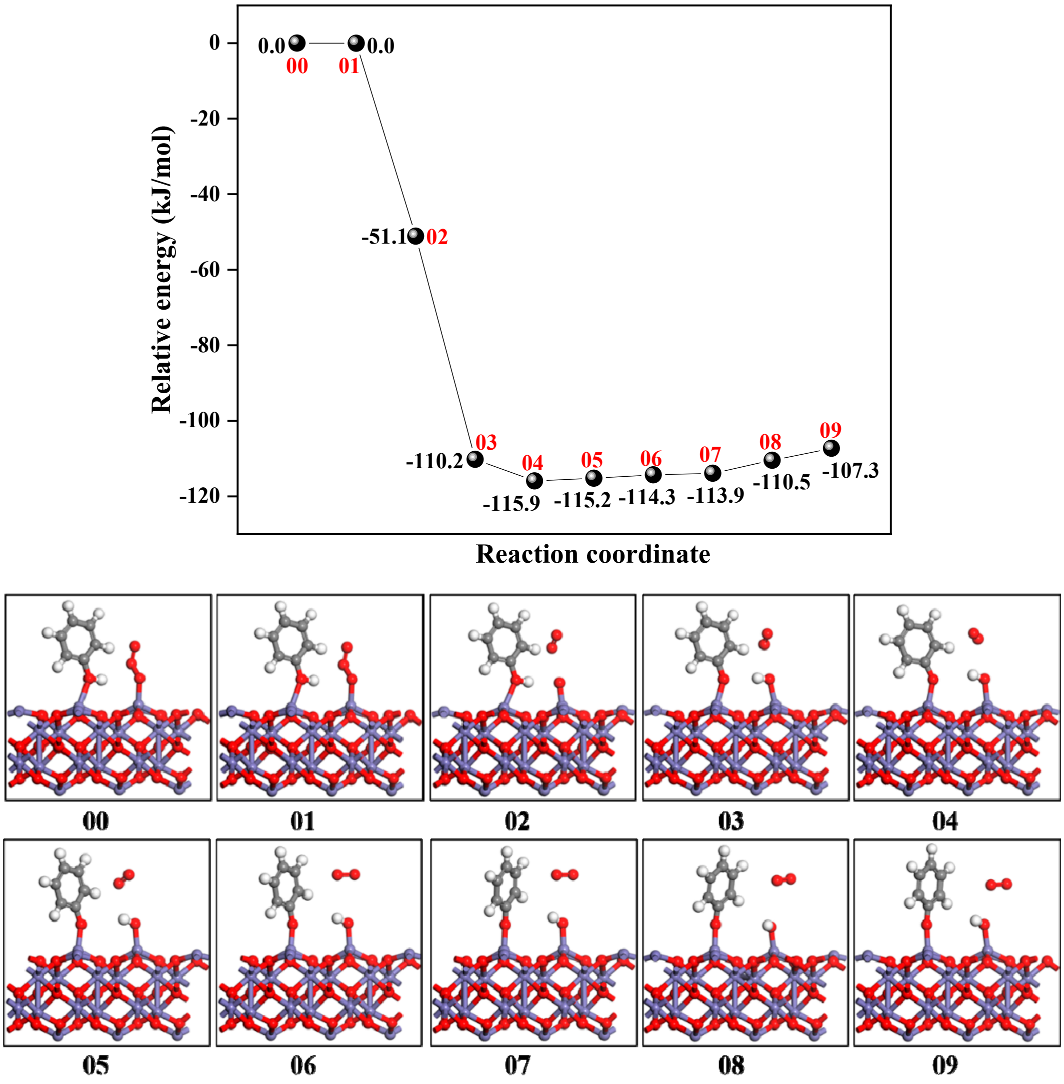

Figure S3 The energy profile for the reaction of C_6H_5OH on the α-Fe_2O_3(0001) surface containing O_3 together with the corresponding structures in one-to-one correspondence with the black points in Figure 2. Energy is in kJ/mol. Color code: Fe (blue), C (gray), O (red), and H (white).

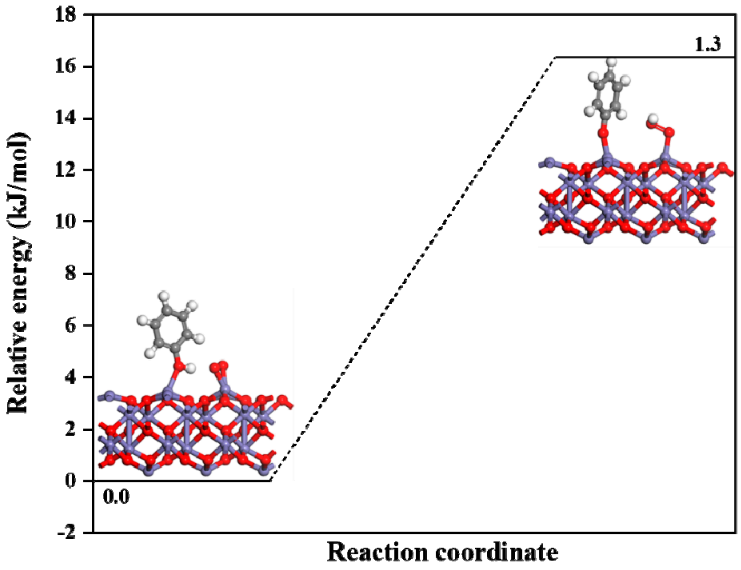

Figure S4 The energy profile for the reaction of C_6H_5OH on the α-Fe_2O_3(0001) surface containing O_2 together with the corresponding structures. Energy is in kJ/mol. Color code: Fe (blue), C (gray), O (red), and H (white).

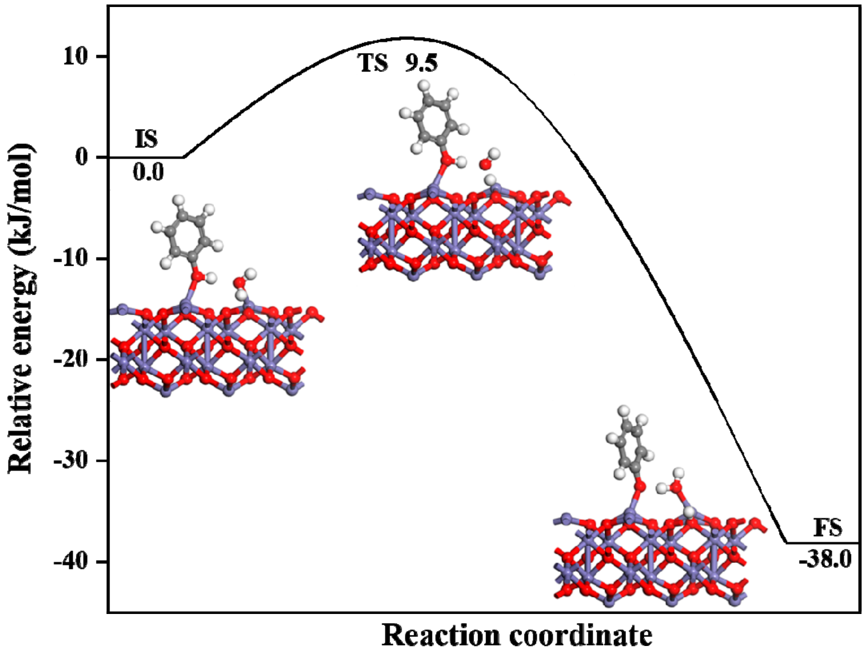

Figure S5 The energy profile for the reaction of C_6H_5OH on the α-Fe_2O_3(0001) surface containing H_2O together with the corresponding structures, including initial state (IS), transition state (TS), and final state (FS). Energy is in kJ/mol. Color code: Fe (blue), C (gray), O (red), and H (white).
